# Mozart’s Music Reduces Fixation Loss in Visual Field Testing

**DOI:** 10.1155/joph/9984011

**Published:** 2026-04-02

**Authors:** Rintra Wongvisavavit, Autsadawut Phoolsawat, Pakin Piriyalukkul, Tunyaluk Panyata, Smatya Wathawathana

**Affiliations:** ^1^ Princess Srisavangavadhana Faculty of Medicine, Chulabhorn Royal Academy, 906 Kamphaeng Phet 6 Rd. Lak Si, Bangkok, Thailand, cra.ac.th; ^2^ Chulabhorn Hospital, Chulabhorn Royal Academy, 906 Kamphaeng Phet 6 Rd. Lak Si, Bangkok, Thailand, cra.ac.th; ^3^ Princess Galyani Vadhana Institute of Music, 2010 Arun Amarin 36, Bang Yi Khan, Bang Phlat, Bangkok, Thailand

**Keywords:** automated perimetry, Mozart, music therapy, reliability of visual field testing, visual field testing

## Abstract

**Purpose:**

This study investigates the impact of Mozart’s music on the reliability of visual field testing among healthy Thai individuals, using a large sample population.

**Methods:**

Automated perimetry was conducted on the right eyes of 163 perimetry‐naive participants using a Humphrey Field Analyzer III (SITA standard 24‐2 program). Participants were randomly assigned to one of three groups: control (no auditory input), headphones (noise‐canceling headphones without music), and Mozart (headphones with Mozart’s Sonata for Two Pianos in D Major, K. 448). Each group received a 10 min pretest intervention according to their group assignment. Key perimetric indices, including fixation loss (FL), false positives (FP), false negatives (FN), test duration, mean deviation (MD), pattern standard deviation (PSD), and sensitivity depression in total and pattern deviation (TD and PD) plots, were collected and analyzed across groups using the Kruskal–Wallis test.

**Results:**

The mean percentages of FL were 14.3% (95% CI 9.2%–19.4%) in the control group, 13.4% (95% CI 7.8%–19%) in the headphones group, and 6.4% (95% CI 4%–8.8%) in the Mozart group. An improvement in FL was observed between the control and Mozart groups (*p* = 0.03). However, no significant differences were found among the groups with respect to FP, FN, or test duration. Additionally, the values for MD, PSD, TD, and PD remained within normal ranges across all groups, with no significant differences.

**Conclusions:**

Mozart’s music may facilitate enhanced concentration and spatial reasoning among individuals undergoing visual field testing with automated perimetry. Consequently, its application in clinical settings has the potential to improve the FL, a reliability index of visual field assessment.

## 1. Introduction

Automated perimetry is the standard tool for assessing visual fields and is commonly employed to measure the visual field in patients with glaucoma or neurological disorders [1]. However, the quality of the visual field test results highly depends on several patient‐related factors, including the individual’s understanding of the testing procedure, adherence to instructions during the test, level of attention and concentration, and emotional states such as anxiety. These factors can compromise test reliability, leading to suboptimal results that may hinder the accurate interpretation and diagnosis of visual field abnormalities [2].

Music has been recognized as a non‐drug treatment method for alleviating behavioral and health problems, and it can help reduce anxiety, pain, and depression in patients [3–7]. Music therapy encompasses various activities, including listening to music, singing, and playing musical instruments. Among these, music listening, referred to as the receptive music therapy method, is one of the most commonly employed approaches for promoting relaxation.

Mozart’s music has been used to explore the reliability of visual field testing in individuals without glaucoma or neurological disorders; listening to Mozart before testing significantly improved the reliability of visual field measurements compared with individuals who did not listen to the music beforehand [8]. Conversely, Shue et al. reported that Mozart’s music did not enhance the reliability of visual field tests in patients with glaucoma [9].

However, a limited number of studies [8–11] have explored the use of Mozart’s music in visual field measurements, and no studies have been conducted among Asian population, necessitating further research for non‐Western cultures. Therefore, the present study considers whether listening to Mozart’s music can improve the reliability of visual field testing among a large cohort of healthy Thai individuals.

## 2. Methods

This prospective, randomized controlled study was approved by the Chulabhorn Royal Academy Institution Review Board (EC 128/2567). Informed consent was obtained from all participants.

The participants between the ages of 20 and 50 who visited the ophthalmology outpatient unit, Chulabhorn Hospital, for annual ophthalmic examinations between December 2024 and April 2025 were asked if they were willing to participate in the study. A total of 163 participants were enrolled after screening. All participants underwent a complete eye examination, including best‐corrected visual acuity (BCVA), refraction, applanation tonometry, slit lamp, and posterior segment measurements. The inclusion criteria for the study were a BCVA score of 20/40 or better, intraocular pressure (IOP) of less than 21 mmHg, spherical refractive error within ±6.00 diopters, cylinder refraction within ±2.00 diopters, and cup‐to‐disc ratio of less than 0.5. The exclusion criteria were any history of ocular surgery, the presence of glaucoma, retinal, or optic nerve diseases, and neurological diseases or intracranial lesions.

The participants were randomized into three groups: the control, headphones, and Mozart groups. All participants were brought to a visual field‐testing room designated for the experiment. In the control group, the participants did not receive headphones or music; they simply remained in the visual field‐testing room for 10 min. The participants assigned to the headphones group received noise‐canceling headphones and wore them for 10 min without listening to music. In the Mozart group, the participants wore headphones and listened to Mozart’s piano sonata for 10 min. The headphones were used to reduce the noise that may disturb the attention of the participants during the test.

Mozart’s Sonata K. 448 was selected for the experiment, specifically the second movement titled Andante. The excerpt extended from the movement’s beginning to measure 48, including the repeat section, resulting in 96 measures. The duration of this excerpt was approximately 5 min per performance. Participants listened to the piece twice consecutively for a total listening time of 10 min. The tempo of the recording was maintained at approximately 56–60 beats per minute, which closely corresponds to the average resting human heartbeat and is considered conducive to relaxation and physiological calm.

All participants underwent automated perimetry using a Humphrey Field Analyzer III (model 860, Carl Zeiss Meditec, Dublin, CA, USA), employing the SITA standard 24‐2 program in the right eye. Only the right eye was evaluated to avoid learning effects and fatigue, which may interfere with the results. The examination procedure was explained by a technician, followed by refractive error correction, and then the perimetry assessment was initiated. The time elapsed between the end of the musical sonata and the conclusion of the visual field assessment was limited to a maximum of 10 min.

Automated perimetry outcomes were collected, including fixation loss (FL), false positive (FP), false negative (FN), test duration, mean deviation (MD), pattern standard deviation (PSD), the number of points depressed at the *p* < 5% level in the total deviation (TD) probability plot, and the number of points depressed at the *p* < 5% level in the pattern deviation (PD) probability plot.

### 2.1. Statistical Analysis

The data of automated perimetry outcomes were presented as the mean with 95% confidence interval (95% CI). The Kruskal–Wallis test was conducted, followed by post hoc Dunn’s test with Bonferroni correction to determine significant differences. A *p* value of < 0.05 was considered significant. All statistical analyses were performed using IBM SPSS statistics 22.0.

## 3. Results

A total of 163 participants were included in the study. Table 1 summarizes the demographic and clinical characteristics of the participants, including sex, age, BCVA, and IOP. The proportion of female participants exceeded half in each group, with 72.2%, 68.5%, and 79.6% in the control, headphones, and Mozart groups, respectively. The median age was 35 years in both the control and headphones groups and 34 years in the Mozart group. Most participants demonstrated a BCVA of 20/20, which was observed in 70.4% of the control and headphones groups and 55.7% of the Mozart group. The median IOP was 13 mmHg in the control group and 14 mmHg in both the headphones and Mozart groups. No significant differences in these baseline characteristics were observed among the groups.

**TABLE 1:** Demographic data.

Characteristics	Control (*n* = 54); *n* (%)	Headphone (*n* = 54); *n* (%)	Mozart (*n* = 54); *n* (%)	*p* value
Sex				0.41
Male	15 (27.8)	17 (31.5)	11 (20.4)	
Female	39 (72.2)	37 (68.5)	43 (79.6)	
Age (years)				
20–29	16 (29.6)	17 (31.5)	16 (29.6)	0.64
30–39	21 (38.9)	23 (42.6)	21 (38.9)	
40–49	13 (24.1)	10 (18.5)	15 (27.8)	
50	4 (7.4)	4 (7.4)	2 (3.7)	
BCVA				0.75
20/20	38 (70.4)	38 (70.4)	29 (53.7)	
20/25	9 (16.7)	12 (22.2)	13 (24.1)	
20/30	5 (9.3)	4 (7.4)	10 (18.5)	
20/40	2 (3.7)	0 (0)	2 (3.7)	
IOP (mmHg)				0.45
10–15	40 (74)	37 (68.5)	45 (83.3)	
16–20	14 (26)	17 (31.5)	9 (16.7)	

*Note:* *p* value: Kruskal–Wallis test, IOP, intraocular pressure.

Abbreviation: BCVA, best‐corrected visual acuity.

**FIGURE 1:**
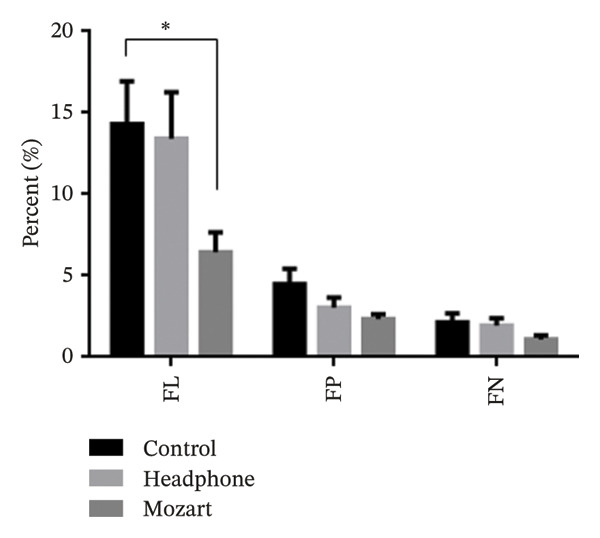
Reliability indices among the control, headphones, and Mozart groups.

**FIGURE 2:**
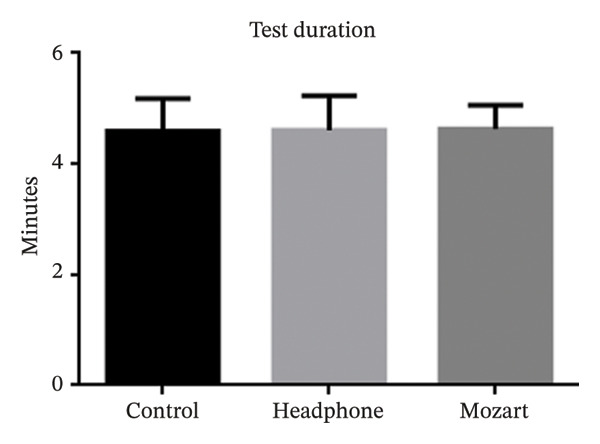
Test duration among the control, headphones, and Mozart groups.

Table 2 summarizes the reliability indices, including the FL, FP, and FN rates, along with the test duration. The mean FL percentages were 14.3% (95% CI 9.2%–19.4%) in the control group, 13.4% (95% CI 7.8%–19%) in the headphones group, and 6.4% (95% CI 4%–8.8%) in the Mozart group. A statistically significant difference in FL was observed between the control and Mozart groups (*p* = 0.03), as illustrated in Figure 1. The mean FP and FN percentages were 4.5% (95% CI 2.6%–6.4%) and 2.2% (95% CI 1.2%–3.2%) for the control group, 3.0% (95% CI 1.7%–4.3%) and 1.9% (95% CI 1%–2.8%) for the headphones group, and 2.3% (95% CI 1.7%–2.9%) and 1.1% (95% CI 0.6%–1.6%) for the Mozart group, respectively. Figure 2 shows the duration of visual field tests among the control, headphones, and Mozart groups. The average test duration was 4.6 min across all groups. No significant differences were found in FP, FN, or test duration among the groups (Figures 1 and 2).

**TABLE 2:** Reliability indices and test duration.

	Control Mean (95% CI)	Headphone Mean (95% CI)	Mozart Mean (95% CI)
Fixation loss (%)	14.3 (9.2–19.4)	13.4 (7.8–19)	6.4 (4–8.8)
False positive (%)	4.5 (2.6–6.4)	3 (1.7–4.3)	2.3 (1.7–2.9)
False negative (%)	2.2 (1.2–3.2)	1.9 (1–2.8)	1.1 (0.6–1.6)
Test duration (minutes)	4.6 (4.4–4.8)	4.6 (4.4–4.8)	4.6 (4.5–4.7)

Automated perimetry results are presented in Table 3. The mean MD values were −0.78 dB in the control group, −0.86 dB in the headphones group, and −0.60 dB in the Mozart group, with corresponding PSD values of 1.74, 1.07, and 1.60 dB, respectively. The mean number of test points with a significance level of *p* < 0.05 in the TD probability plot was 5.7, 5.9, and 5.7 in the control, headphones, and Mozart groups, respectively. Similarly, for the PD probability plot, the number of significantly depressed points was 6.0, 5.3, and 5.2 in the control, headphones, and Mozart groups, respectively. No significant differences were observed in any of the perimetry parameters across the three groups.

**TABLE 3:** Evaluation of automated perimetry outcomes across the three groups.

	Control Mean (95% CI)	Headphone Mean (95% CI)	Mozart Mean (95% CI)	*p* value
MD (dB)	−0.78 (−1–−0.5)	−0.86 (−1.2–−0.6)	−0.6 (−1–−0.2)	0.41
PSD (dB)	1.74 (1.6–1.9)	1.07 (0.9–1.2)	1.6 (1.5–1.7)	0.34
Points *p* < 5% (total deviation)	5.7 (4.2–7.2)	5.9 (4.2–7.6)	5.7 (3.9–7.5)	0.70
Points *p* < 5% (pattern deviation)	6 (5.2–7.6)	5.3 (4.2–6.4)	5.2 (4.2–6.2)	0.63

Abbreviations: MD, mean deviation; PSD, pattern standard deviation.

## 4. Discussion

Automated perimetry is an effective method for the detection, diagnosis, and longitudinal monitoring of various ocular and neurological disorders, with particular significance in managing glaucoma. The reliability of visual field assessments is a critical determinant of test quality. Inadequate reliability may result in inaccurate or misleading outcomes, thereby impairing the clinician’s ability to identify pathological changes and accurately evaluate disease progression.

The reliability of visual field assessments is influenced by multiple factors, including the patient’s comprehension of the testing procedure, compliance with instructions, and level of attention, as well as their emotional state, such as feeling depressed or anxious [2]. The main reliability indices used in evaluating automated perimetry are FL, FP, and FN. These indices provide essential insights into the validity and accuracy of the visual field test, ensuring that clinical decisions are based on dependable data. According to the criteria recommended by Humphrey Instruments, Inc. (San Leandro, CA), a reliable test is defined as less than 20% FL, with FP and FN error rates below 33% [12]. FL occurs when a participant reports seeing a stimulus within the physiological blind spot, suggesting compromised fixation. Previous studies have identified FL as the most frequent indicator of unreliable visual field results, as it is highly dependent on patient concentration [13, 14]. An FP response is recorded when the participant reacts in the absence of a stimulus, whereas an FN response is recorded when the participant fails to respond to stimuli that probably should have been seen. Collectively, these indices serve as proxies for the subject’s vigilance and sustained concentration during testing.

The FL, FP, and FN percentages in our study were within the standard range in all groups. It could be assumed that there was no clinical difference between groups. However, an improvement in the percentages of FL in the Mozart group compared to the control group may be attributed to the Mozart effect, which improves spatial reasoning for a short duration (i.e., within 10 min). Specifically, Rauscher et al. demonstrated that Mozart’s K. 448 improves spatial–temporal performance [15]. Furthermore, Mozart’s K. 448 has been shown to enhance short‐term memory and concentration, potentially through activation of cortical regions involved in attention, memory, and sensory–motor integration [16, 17], as well as limbic structures associated with emotional processing [18, 19].

The second movement (Andante) of K.448, applied in this study, characterized by a slow, regular tempo and harmonic stability, creates a calming auditory environment that has been associated with neurophysiological changes [20], including modulation of EEG activity and improved cognitive focus [21–23]. These musical and neural properties are likely contributors to the cognitive and emotional benefits observed and may explain the reduction in FL noted in the present study.

Improvement of FL was also found in a previous study that compared the effect of Mozart’s music on the reliability of visual field tests in a healthy population with a mean age of 23 years [8]. Significant reductions in FL, FP, and FN were observed for the group that listened to the music compared with the control group. It was assumed that Mozart’s music improved spatial–temporal reasoning. In contrast, Shue et al. demonstrated the effect of Mozart’s music on the reliability of visual field tests in glaucomatous patients with a mean age of 65 years, showing that there were no significant differences in FL, FP, and FN among the music‐listening, headphones, and control groups [9]. This may be because the elderly glaucomatous patients developed neurodegeneration, which affected spatial–temporal reasoning.

Concerning FP and FN responses, FN values have been correlated with the severity of visual field impairment; in contrast, FP responses are often attributed to “trigger‐happy” behavior, wherein participants respond in the absence of a stimulus [24]. Elevated FP rates are also frequently associated with higher MD values [25, 26]. Our results showed that the FP and FN values remained within the normal limits across all groups, with no significant differences between them. Furthermore, there was no evidence of visual field defects in any group, with comparable values observed across the MD, PSD, TD, and PD parameters. Meanwhile, the mean test duration was approximately 4.6 min across all groups, aligning with the expected testing time for adults, which is typically less than 5 min [27]. Thus, the testing duration was consistent across the groups and fell within the normal range.

To the best of our knowledge, this study represents the first investigation into the effects of Mozart’s music on automated perimetry performance in an Asian population. The reliability outcomes observed in this study are attributable to the direct influence of Mozart’s music, rather than confounding factors such as learning effects or fatigue. Previous research has shown that repeated exposure to visual field testing, particularly after three or more sessions, can enhance test reliability owing to the learning effect [14]. To minimize this bias, we recruited participants with no prior experience in visual field testing. In addition, data collection was limited to the right eye to reduce the potential impact of fatigue associated with bilateral testing. These methodological considerations constitute the key strengths of the present study.

Nonetheless, this study has several limitations. The participant cohort consisted exclusively of individuals under the age of 50, which may not adequately represent the typical patient populations undergoing visual field testing, such as those with glaucoma or neurological disorders. Similarly, this study focused on a particular Asian population, which may not represent all Asian demographics. Therefore, future research should be conducted to enhance the generalizability and clinical applicability of the findings.

### 4.1. Future Perspectives

Music exerts various effects on brain function, potentially enhancing concentration, regulating emotional states, and reducing anxiety. In the present study, Mozart’s K. 448 was selected because of its well‐documented positive influence on attention and emotional regulation. It is hypothesized that other musical compositions with comparable tempo, rhythm, and harmonic structure may yield similar effects and could be explored in future research. Moreover, subsequent studies may consider incorporating culturally relevant, participant‐selected, and relaxing music to enhance individual engagement and satisfaction. Regarding direct assessment of attentional engagement during visual field testing, the incorporation of gaze tracking or eye position variability analyses would enable direct evaluation of eye movements and may provide additional physiological insight into fixation stability and attention.

Future investigations are also needed in clinical populations, particularly individuals with glaucoma, who represent a primary demographic undergoing automated perimetry. The incorporation of music in such settings may contribute to improve test reliability and patient experience. Further studies are needed to accumulate additional evidence and verify these findings and hypotheses in both broader and more specific study populations.

## 5. Conclusion

Listening to Mozart’s Sonata for Two Pianos in D Major, K. 448 was found to enhance the FL, one of the reliability indices of visual field assessments conducted using automated perimetry. This may be attributed to the music’s ability to stimulate temporal–spatial reasoning and increase participant concentration. These findings should be considered hypothesis‐generating and suggest that music may have potential as an adjunctive approach to support test performance in clinical settings. However, future studies in neurodegeneration patients, such as glaucoma is required.

## Author Contributions

Rintra Wongvisavavit is the guarantor of the study, supervised the work, and administered the project. Rintra Wongvisavavit and Smatya Wathawathana conceptualized the study, drafting, and editing the manuscript. Autsadawut Phoolsawat, Pakin Piriyalukkul, and Tunyaluk Panyata performed automated perimetry and collected data. Rintra Wongvisavavit interpreted and analyzed the data.

## Funding

This research project was funded by the Chulabhorn Royal Academy (Project code RF‐CRA‐2568‐001).

## Disclosure

All authors read and approved the final manuscript. The funding organization had no role in the study design, data collection, data analysis, decision to publish, or preparation of the manuscript.

## Ethics Statement

This study was approved by the Chulabhorn Royal Academy Institution Review Board (EC 128/2567). All the research procedures were conducted in accordance with the principles of the Declaration of Helsinki.

## Consent

Written informed consent was obtained from all participants.

## Conflicts of Interest

The authors declare no conflicts of interest.

## Data Availability

The data that support the findings of this study are available upon request to the corresponding authors.

## References

[1] Quigley H. A. , Glaucoma, Lancet. (2011) 377, no. 9774, 1367–1377, 10.1016/s0140-6736(10)61423-7, 2-s2.0-79954621581. 21453963

[2] Chew S. S. , Kerr N. M. , Wong A. B. , Craig J. P. , Chou C. Y. , and Danesh-Meyer H. V. , Anxiety in Visual Field Testing, British Journal of Ophthalmology. (2016) 100, no. 8, 1128–1133, 10.1136/bjophthalmol-2015-307110, 2-s2.0-84982787200. 26608027

[3] Lu G. , Jia R. , Liang D. , Yu J. , Wu Z. , and Chen C. , Effects of Music Therapy on Anxiety: A Meta-Analysis of Randomized Controlled Trials, Psychiatry Research. (2021) 304, 10.1016/j.psychres.2021.114137. 34365216

[4] Lee J. H. , The Effects of Music on Pain: A Meta-Analysis, Journal of Music Therapy. (2016) 53, no. 4, 430–477, 10.1093/jmt/thw012, 2-s2.0-85014098116. 27760797

[5] Zhao K. , Bai Z. G. , Bo A. , and Chi I. , A Systematic Review and Meta-Analysis of Music Therapy for the Older Adults With Depression, International Journal of Geriatric Psychiatry. (2016) 31, no. 11, 1188–1198, 10.1002/gps.4494, 2-s2.0-84964319974. 27094452

[6] Rodgers-Melnick S. N. , Lin L. , Gam K. et al., Effects of Music Therapy on Quality of Life in Adults With Sickle Cell Disease (Musiqols): A Mixed Methods Feasibility Study, Journal of Pain Research. (2022) 15, 71–91, 10.2147/jpr.S337390. 35046718 PMC8760983

[7] Dahshan D. , Kuzbel J. , and Verma V. , A Role for Music in Cataract Surgery: A Systematic Review, International Ophthalmology. (2021) 41, no. 12, 4209–4215, 10.1007/s10792-021-01986-9. 34312781

[8] Fiorelli V. M. B. , Kasahara N. , Cohen R. et al., Improved Automated Perimetry Performance Following Exposure to Mozart, British Journal of Ophthalmology. (2006) 90, no. 5, 543–545, 10.1136/bjo.2005.085902, 2-s2.0-33646255098. 16481380 PMC1857072

[9] Shue B. , Chatterjee A. , Fudemberg S. et al., The Effects of Mozart’s Music on the Performance of Glaucoma Patients on Automated Perimetry, Investigative Ophthalmology and Visual Science. (2011) 52, no. 10, 7347–7349, 10.1167/iovs.11-7430, 2-s2.0-84856407815. 21828156

[10] Marques J. C. , Vanessa A. C. , Fiorelli M. B. , and Kasahara N. , Improved Automated Perimetry Performance in Elderly Subjects After Listening to Mozart, Clinics (Sao Paulo). (2009) 64, no. 7, 665–667, 10.1590/s1807-59322009000700010, 2-s2.0-69149093982. 19606243 PMC2710440

[11] Gall C. , Geier J. S. , Sabel B. A. , and Kasten E. , Does Music Influence Visual Perception in Campimetric Measurements of the Visual Field?, Psychotherapie, Psychosomatik, Medizinische Psychologie. (2009) 59, no. 1, 31–37, 10.1055/s-2007-986318, 2-s2.0-65349194476. 18240114

[12] Birt C. M. , Shin D. H. , Samudrala V. , Hughes B. A. , Kim C. , and Lee D. , Analysis of Reliability Indices From Humphrey Visual Field Tests in an Urban Glaucoma Population, Ophthalmology. (1997) 104, no. 7, 1126–1130, 10.1016/s0161-6420(97)30173-0, 2-s2.0-0030758978. 9224465

[13] Kumar A. , Hekmatjah N. , Yu Y. , Han Y. , Ying G. S. , and Oatts J. T. , Factors Associated With Visual Field Testing Reliability in Children With Glaucoma or Suspected Glaucoma, American Journal of Ophthalmology. (2024) 264, 187–193, 10.1016/j.ajo.2024.04.005. 38614194 PMC11257782

[14] Tiwari U. S. , Aishwarya A. , and Bhale A. , Influence of Learning Effect on Reliability Parameters and Global Indices of Standard Automated Perimetry in Cases of Primary Open Angle Glaucoma, Romanian Journal Ophthalmology. (2018) 62, no. 4, 277–281, 10.22336/rjo.2018.42. PMC6421494 30891523

[15] Rauscher F. H. , Shaw G. L. , and Ky K. N. , Music and Spatial Task Performance, Nature. (1993) 365, no. 6447, 10.1038/365611a0, 2-s2.0-0027451909. 8413624

[16] Limyati Y. , Wahyudianingsih R. , Maharani R. , and Christabella M. T. , Mozart’s Sonata for Two Pianos K. 448 in D-Major 2nd Movement Improves Short-Term Memory and Concentration, Journal of Medicine and Health. (2019) 2, no. 4, 10.28932/jmh.v2i4.1127.

[17] Warren J. D. , Uppenkamp S. , Patterson R. D. , and Griffiths T. D. , Separating Pitch Chroma and Pitch Height in the Human Brain, Proceedings of the National Academy of Sciences of the USA. (2003) 100, no. 17, 10038–10042, 10.1073/pnas.1730682100, 2-s2.0-0041689888. 12909719 PMC187755

[18] Vuust P. , Heggli O. A. , Friston K. J. , and Kringelbach M. L. , Music in the Brain, Nature Reviews Neuroscience. (2022) 23, no. 5, 287–305, 10.1038/s41583-022-00578-5. 35352057

[19] Warren J. , How Does the Brain Process Music?, Clinical Medicine. (2008) 8, no. 1, 32–36, 10.7861/clinmedicine.8-1-32, 2-s2.0-39149116723. 18335666 PMC4953706

[20] Shi W. Y. , Re-Examining the Mozart Effect: The Sonata in D Major, K. 448 and the Influence of Rhythm on Spatial Intelligence, Journal of Multidisciplinary Research. (2020) 12, no. 2, 121–129.

[21] Ding R. , Tang H. , Liu Y. et al., Therapeutic Effect of Tempo in Mozart’s “Sonata for Two Pianos” (K. 448) in Patients With Epilepsy: An Electroencephalographic Study, Epilepsy and Behavior. (2023) 145, 10.1016/j.yebeh.2023.109323. 37356223

[22] Lin L. C. , Ouyang C. S. , Chiang C. T. , Wu R. C. , Wu H. C. , and Yang R. C. , Listening to Mozart K. 448 Decreases Electroencephalography Oscillatory Power Associated With an Increase in Sympathetic Tone in Adults: A Post-Intervention Study, JRSM Open. (2014) 5, no. 10, 10.1177/2054270414551657. PMC4221897 25383198

[23] Guo X. , Wang C. , and Guo J. , The Effect of Mozart’s K. 448 on Epilepsy: A Systematic Literature Review and Supplementary Research on Music Mechanism, Epilepsy and Behavior. (2025) 163, 10.1016/j.yebeh.2024.110108. 39637732

[24] Heijl A. and Patella V. M. , Essential Perimetry: The Field Analyzer Primer, 2002, Carl Zeiss Meditec.

[25] Tan N. Y. , Tham Y.-C. , Koh V. et al., The Effect of Testing Reliability on Visual Field Sensitivity in Normal Eyes: The Singapore Chinese Eye Study, Ophthalmology. (2018) 125, no. 1, 15–21, 10.1016/j.ophtha.2017.08.002, 2-s2.0-85028474877. 28863943

[26] Yohannan J. , Wang J. , Brown J. et al., Evidence-Based Criteria for Assessment of Visual Field Reliability, Ophthalmology. (2017) 124, no. 11, 1612–1620, 10.1016/j.ophtha.2017.04.035, 2-s2.0-85021441017. 28676280 PMC5675138

[27] Kahook M. Y. and Noecker R. J. , How Do You Interpret a 24-2 Humphrey Visual Field Printout, Glaucoma Today. (2007) 1, 57–62.

